# Comparison of Transcutaneous Electrical Nerve Stimulation and Parasternal Block for Postoperative Pain Management after Cardiac Surgery

**DOI:** 10.1155/2016/4261949

**Published:** 2016-04-12

**Authors:** Nilgun Kavrut Ozturk, Elif Dogan Baki, Ali Sait Kavakli, Ayca Sultan Sahin, Raif Umut Ayoglu, Arzu Karaveli, Mustafa Emmiler, Kerem Inanoglu, Bilge Karsli

**Affiliations:** ^1^Department of Anesthesiology and Reanimation, Antalya Education and Research Hospital, Varlık Mahallesi, Kazim Karabekir Cadde, 07100 Antalya, Turkey; ^2^Department of Anesthesiology and Reanimation, Afyon Kocatepe University, Faculty of Medicine, Afyon, Turkey; ^3^Department of Cardiovascular Surgery, Antalya Education and Research Hospital, 07100 Antalya, Turkey; ^4^Department of Algology, Akdeniz University, Faculty of Medicine, 07100 Antalya, Turkey

## Abstract

*Background*. Parasternal block and transcutaneous electrical nerve stimulation (TENS) have been demonstrated to produce effective analgesia and reduce postoperative opioid requirements in patients undergoing cardiac surgery.* Objectives*. To compare the effectiveness of TENS and parasternal block on early postoperative pain after cardiac surgery.* Methods*. One hundred twenty patients undergoing cardiac surgery were enrolled in the present randomized, controlled prospective study. Patients were assigned to three treatment groups: parasternal block, intermittent TENS application, or a control group.* Results*. Pain scores recorded 4 h, 5 h, 6 h, 7 h, and 8 h postoperatively were lower in the parasternal block group than in the TENS and control groups. Total morphine consumption was also lower in the parasternal block group than in the TENS and control groups. It was also significantly lower in the TENS group than in the control group. There were no statistical differences among the groups regarding the extubation time, rescue analgesic medication, length of intensive care unit stay, or length of hospital stay.* Conclusions*. Parasternal block was more effective than TENS in the management of early postoperative pain and the reduction of opioid requirements in patients who underwent cardiac surgery through median sternotomy. This trial is registered with Clinicaltrials.gov number NCT02725229.

## 1. Introduction

It has been previously reported that the mortality and morbidity of cardiac surgery may increase due to cardiovascular, pulmonary, renal, and infectious complications. To decrease these complications, recovery times should be reduced and neuroendocrine stress responses to surgery should be blunted [1]. Patients experiencing pain after undergoing cardiac surgery may also experience prolonged immobilization, insufficient respiratory functions, and the inability to cough due to median sternotomy. Therefore, duration of mechanical ventilation, length of intensive care unit (ICU) stay, and length of hospital stay of these patients may increase significantly. Therefore, early tracheal extubation should be facilitated with the use of effective analgesic methods and drugs [2, 3]. Invasive and noninvasive interventions, such as epidural analgesia, local regional blockade, and the use of intravenous (IV) opioids, are used for postoperative pain management [4]. IV opioids are often used for postoperative analgesia; however, undesired side effects, such as respiratory depression, profound sedation, and nausea/vomiting, may delay tracheal extubation when administered at doses necessary to provide pain relief [5, 6]. Opioids may also lead to cough suppression and drowsiness and limit effective expectoration.

A major source of pain in patients undergoing cardiac surgery is the median sternotomy incision and the mediastinal tube sites. The anterior and posterior branches of intercostal nerves innervate the sternum. Parasternal local anesthetic infiltration around the sternum has been demonstrated to be useful in providing early postoperative analgesia, reducing opioid requirements, and, therefore, producing a potential positive effect on recovery. It is a simple and rapid technique that can be used even for anticoagulated patients [5, 7].

Transcutaneous electrical nerve stimulation (TENS) is a noninvasive technique that is effective for postoperative pain management [8, 9]. It has been demonstrated to produce effective analgesia and reduce postoperative opioid requirements in patients undergoing cardiac surgery, and it has no side effects [10]. It is a comfortable, noninvasive, and nonpharmacological method, which can be easily performed. To date, we have not found any published studies comparing the analgesic effects of TENS and parasternal block in patients undergoing cardiac surgery through median sternotomy. Therefore, the objective of the present study was to compare the efficacy of TENS and parasternal block with local anesthetic infiltration in relieving pain during the first 24 h period following median sternotomy.

## 2. Methods

The present, prospective, randomized, controlled study included 120 patients, 18 to 65 years of age, who were scheduled for elective valve repair or coronary artery bypass graft (CABG) surgery with cardiopulmonary bypass. All patients provided informed signed consent and the permission of the human ethics board of the hospital was obtained. Criteria for exclusion included previous sternotomy for CABG or heart valve surgery; emergency surgery; ejection fraction <40%; congestive heart failure; an allergy to amide-based local anesthetics, opioids, or benzodiazepines; inability to provide informed consent; prolonged cardiopulmonary bypass time (>145 min); and previous experience with TENS. Postoperative exclusion criteria included patients requiring intra-aortic balloon pump, additional surgery, or emergency surgery.

All patients included in the present study were informed about the visual analogue scale (VAS) and the use of an IV patient-controlled analgesia (PCA) device during the preanesthetic examination.

A random number table was used to randomly allocate patients to one of three treatment groups to relieve postoperative pain during the first 24 h following median sternotomy: parasternal block group (parasternal block combined with levobupivacaine infiltration and PCA), TENS group (TENS and PCA), or the control group (PCA alone). All patients were premedicated with oral diazepam (0.2 mg/kg) the night before the surgery. In the operating room, an 18-gauge IV catheter and a 20-gauge radial arterial cannula were inserted. After monitoring electrocardiography and invasive arterial pressures, anesthesia was induced using 5 *μ*g/kg fentanyl and 0.3 mg/kg to 0.6 mg/kg etomidate, while the intubation was facilitated using 0.6 mg/kg to 1.0 mg/kg rocuronium. Anesthesia was maintained with 1% to 2% sevoflurane in 50% oxygen and 50% air and 5 *μ*g/kg/h to 10 *μ*g/kg/h fentanyl infusion; IV rocuronium was administered to maintain the train-of-four (TOF) monitoring count at 1 or 2. A triple-lumen central venous catheter (Certofix Duo, B Braun Melsungen AG, Germany) was introduced into the right internal jugular vein. Intraoperative monitoring included electrocardiography, invasive arterial blood pressure, pulse oximetry, end-tidal carbon dioxide, and serial arterial blood gas analysis. Central venous pressure, central temperature, and urine output were also continuously monitored. During cardiopulmonary bypass, oxygenation was achieved using a membrane oxygenator and sevoflurane was administered using the oxygenator at a minimum alveolar concentration of 1.0.

Before sternal wire placement, sternotomy and mediastinal tube sites were infiltrated with a solution of 25 mL levobupivacaine and 25 mL saline (total of 50 mL) mixture (parasternal block group) for 40 patients. The study solution was administered in 2 mL aliquots injected into five anterior (2nd to 6th) intercostal spaces on each side of the sternum and 20 mL over the periosteum, while the entrance of chest tubes was deeply infiltrated with 10 mL. All patients in the TENS group and control group received parasternal intercostal block with saline injected in the same volume as levobupivacaine.

At the conclusion of the surgery, all anesthetics were discontinued and patients were transferred to the ICU where they were connected to a mechanical ventilator. Arterial oxygen saturation, invasive arterial pressures, and central venous pressures were monitored. All patients were given 1 g of acetaminophen at the conclusion of surgery and 2 mg IV morphine on the arrival to the ICU to receive postoperative analgesia before administration of PCA. The patients were extubated on the condition that they were awake and responsive to commands, fully warmed to a core temperature >36°C, hemodynamically stable without significant dysrhythmias, well perfused with adequate urine output (>1.0 mL/kg/h), mediastinal bleeding <100 mL/h, respiratory rate between 10 breaths/min and 30 breaths/min, oxygen saturation >95% with 50% oxygen, pH >7.30, and a VAS score ≤5. The time from the ICU admission to extubation was defined as “extubation time.” All of the patients included in the present study started to receive morphine infusions with an IV PCA device (Abbott Pain Manager II, Abbott Laboratories, USA) for postoperative analgesia as they were admitted to the ICU. The PCA device was set to deliver a bolus dose of 2 mg with a lockout interval of 12 min and with a maximum 4 h limit of 40 mg for every patient. All patients in the parasternal block group received placebo TENS as described below.

A Biomed Plus TENS device (Bio Medical Life Systems, USA) was used for therapeutic and placebo stimulation. Therapeutic TENS unit produced an asymmetrical square biphasic waveform at a frequency of 100 pulses/s and a pulse width of 100 *μ*s. Two electrodes (first unit channel) were placed on one side of the incision and two other electrodes (second unit channel) were placed on the other side. The electrodes (5 × 5) were positioned 1 cm away from the wound closure line. Electrical stimulation for 1 h was followed by a 1 h rest interval; 1 h stimulation was then repeated. The stimulus intensity was adjusted until a strong, but comfortable, tingling sensation was experienced. The internal programming of the placebo stimulation device was modified to reduce the rest time between the pulses from 350 ms to 33 sec with a view to preventing analgesic effect.

Patients in the control group (*n* = 40 patients) received parasternal block with saline, placebo TENS, and PCA according to the same protocol.

All patients were given an additional 50 mg IV tramadol when they required rescue analgesia beyond their PCA lockout.

Pain intensity was assessed 4 h, 5 h, 6 h, 7 h, 8 h, 12 h, and 24 h postoperatively in the ICU using a VAS, which ranged from 0 (no pain) to 10 (unbearable pain). If the patients were still intubated, the observer asked whether he or she had a pain score of 10 points; if this was not the case, the observer repeated the question decreasing the score 1 point each time, until the patient confirmed the answer by nodding. If the patient's conscious state did not allow pain evaluation, his or her data were removed from analysis. The investigators who assessed the postoperative VAS scores were blind to the method used for analgesia.

Morphine delivered via the PCA pump in the first 24 h postoperatively, mean arterial pressure (MAP), heart rate, and oxygen saturation were simultaneously recorded. Quality of oxygenation was assessed using arterial blood gas analysis (on patient's arrival to the ICU, at extubation, and at 6 h and 12 h postoperatively), which consisted of the partial pressure of CO_2_, pH, the partial pressure of O_2_, and calculation of the partial pressure of O_2_ to fraction of inspired oxygen ratio. Age, sex, body mass index, length of extubation time, length of ICU stay, length of hospital stay, rescue analgesics intake, and any opioid-related side effects (such as nausea/vomiting, constipation) were also recorded for each patient. Postoperative nausea and vomiting were treated with 4 mg IV ondansetron, as required.

It was calculated that a sample size of 33 patients in each group would have 90% power to detect a 20% difference in the VAS between the control group and the other groups; therefore, 40 patients per group were included to replace any dropouts. Statistical analyses were performed using SPSS version 16.0 (IBM Corporation, USA). For continuous variables, the unpaired Student's *t*-test was used to compare the groups at each time point of data collection. Repeated measures ANOVA was used to compare these variables across the 24 h time period. When a statistically significant interaction was found among the groups, the Greenhouse-Geisser correction was applied to detect the exact location. Data were expressed as the mean (±SD) where indicated. *P* < 0.05 was considered to be statistically significant.

## 3. Results

A total of 120 patients were enrolled in the present study; however, two patients from the parasternal block group and three patients from the control group were excluded due to excessive bleeding and the need for additional surgery.

Table 1 summarizes the demographic and perioperative characteristics of the patients. There were no statistically significant differences among groups.

**Figure 1:**
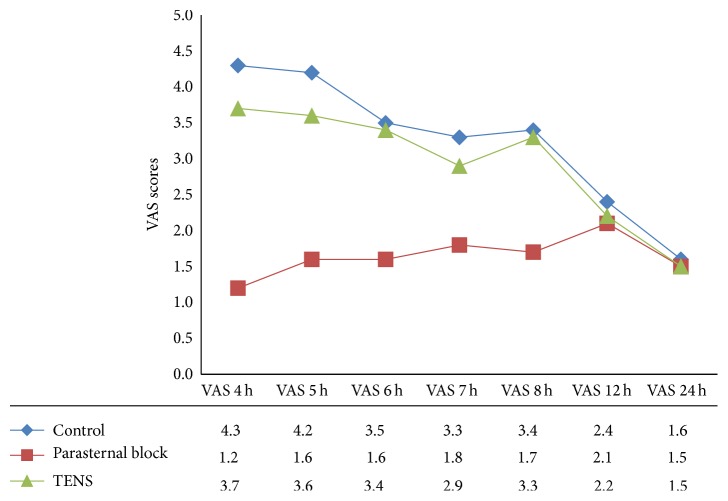
Changes in the visual analogue scale (VAS) scores in the control, parasternal block, and the transcutaneous electrical nerve stimulation (TENS) groups within the first 24 h postoperatively (*P* < 0.001 for 4 h, 5 h, 6 h, 7 h, and 8 h).

The mean VAS scores were significantly lower in the parasternal block group than in the control and TENS groups at 4 h, 5 h, 6 h, 7 h, and 8 h postoperatively (*P* < 0.001). The differences at 12 h and 24 h were not significant. Although VAS scores were lower in the TENS group compared with the control group at most of the time intervals, the differences were not significant (Figure 1).

The main postoperative outcomes such as MAP, extubation time, length of ICU stay, length of hospital stay, morphine consumption, and total tramadol consumption are presented in Table 2. The mean (±SD) morphine doses administered during the postoperative 24 h period were 52.4 ± 23.1 mg, 26.5 ± 13.3 mg, and 37 ± 15.2 mg in the control, parasternal block, and TENS group, respectively. Morphine requirements were significantly lower in the parasternal block group than in the TENS and control group after surgery (*P* < 0.001). It was also significantly lower in the TENS group than in the control group (*P* < 0.001) (Table 2).

There were no statistical differences among the groups regarding the extubation time, length of ICU stay, or length of hospital stay. There were no statistically significant differences among the groups with respect to the rescue analgesic medication.

No significant differences were observed between oxygen saturation and MAP during the postoperative period. Similarly, no major effect according to treatment group was observed in the arterial blood gases. No neurological dysfunction was observed in any patient included in the present study throughout the study period.

No local anesthetic-related (such as local anesthetic toxicity, allergic reactions) or opioid-related (such as severe nausea/vomiting, respiratory depression, and constipation) side effects were observed.

In the first three months, there were no differences among the groups regarding wound complications such as infection or dehiscence.

## 4. Discussion

Elective CABG may lead to pain due to median sternotomy and chest tube entrance incisions. These patients may also experience immobility, insufficient ventilation, and inability to cough. Furthermore, weaning from mechanical ventilation may be delayed and the length of ICU and hospital stay may be prolonged. Therefore, accelerating extubation with effective analgesic methods and drugs is the main target for both surgeons and anesthesiologists.

In the present study, we compared the effectiveness of the parasternal block using levobupivacaine and TENS in relieving poststernotomy pain. To our knowledge, the present randomized study was the first to compare the effects of TENS and parasternal block using levobupivacaine on pain after median sternotomy for CABG surgery. However, there are studies that have investigated the effect of parasternal block using local anesthetic infiltration or TENS alone for postoperative pain. Infiltration of median sternotomy and mediastinal tube sites using 60 mL of 0.25% levobupivacaine resulted in a decrease in morphine consumption within 24 h postoperatively in the study conducted by Kocabas et al. [7]. However, they did not find any difference in VAS scores 1 h, 2 h, 3 h, 4 h, 8 h, 12 h, 18 h, and 24 h postoperatively between the parasternal block and control groups. On the contrary, in our study we found significantly lower VAS scores 4 h to 8 h postoperatively. The conflicting results may be due to different assessment times. We did not assess VAS scores in the first 3 h postoperatively because the patients would possibly still be intubated and under the influence of anesthetic/analgesic agents. McDonald et al. [5] also found lower morphine consumption and similar VAS scores in their study when they compared parasternal levobupivacaine versus placebo infiltration. This may be due to early extubation in the operating room because it could complicate the patients' perception of pain or the small sample size (*n* = 17) used in their study.

While previous studies have reported better postoperative respiratory parameters using parasternal blockade [5, 7], others demonstrated no improvement in respiratory functions [11]. Similarly, we failed to demonstrate any improvement in postoperative respiratory functions and oxygenation. We believe this may be explained by the suggestion that sternotomy pain was just a minor factor in the respiratory dysfunction observed following cardiac surgery.

There are several studies demonstrating that TENS is effective in controlling poststernotomy and postthoracotomy pain [12–14]. However, some studies failed to demonstrate any superiority of TENS over pharmacological analgesia for postthoracotomy pain [15, 16]. In a recent meta-analysis of randomized trials, Sbruzzi et al. [17] concluded that TENS provided additional pharmacological analgesia because it promoted greater pain relief compared with placebo TENS in patients after thoracic surgery. Emmiler et al. [12] found that TENS was more effective than placebo TENS or control treatments in decreasing pain and limiting the intake of opioid and nonopioid medication within the first 24 h following median sternotomy.

In the present study, although the VAS scores were lower in the TENS group compared with the control group, the difference was not significant. It is believed that this may be due to relatively short duration of TENS protocol (a total of 12 h) or the intermittent application of TENS.

The groups did not differ with respect to the length of hospital stay or ICU stay. This may be due to strict discharge criteria used in the cardiovascular ICU at our institute. Our findings were consistent with other studies that demonstrated no benefit from TENS with respect to the length of hospital stay or the length of ICU stay [15, 18].

There are several analgesic methods, such as IV opioids, intrathecal morphine, and epidural analgesia, used in CABG surgery. Opioids are one of the most popular analgesic agents administered to patients after cardiac surgery; however, it is difficult to determine the minimum effective concentration. Although they relieve pain, they may also lead to cough suppression, drowsiness, nausea, and vomiting and limit effective expectoration. In addition, opioids are associated with many side effects including respiratory depression, sedation, urinary retention, and constipation. Although epidural analgesia may be an effective method, many anesthesiologists believe that the risk for potential epidural hematoma may outweigh its benefits. There is also a trend toward using alternative pain management methods for postoperative pain. In the current study, we compared the effectiveness of TENS and parasternal block using levobupivacaine, and we suggest that parasternal block is superior to TENS and may be a viable alternative to pharmacological analgesics for the treatment of median sternotomy pain.

## 5. Conclusion

Although it decreased postoperative opioid requirements, TENS was not found to be as effective as parasternal block for the management of postoperative pain after median sternotomy for CABG. Parasternal block reduced pain scores better than postoperative intermittent TENS protocol for the first 4 h to 8 h postoperatively. This method is simple and quickly performed and, unlike neuroaxial blocks, it can be used in patients who are anticoagulated perioperatively.

## Figures and Tables

**Table 1:** Demographic and perioperative characteristics.

Characteristic	Treatment group			*P*
	Control (*n* = 37)	Parasternal block (*n* = 38)	TENS (*n* = 40)	
Age, years	60.1 ± 12.6	63.5 ± 10.2	58.2 ± 11.5	0.438
Sex, *n*				
Female	13	13	13	0.909
Male	24	25	27	
Weight, kg	76.1 ± 11.3	70.6 ± 10.8	75 ± 14.1	0.128
Height, cm	167 ± 9	169 ± 8	170 ± 9	0.228
Ejection fraction, %	52 ± 4	55 ± 6	55 ± 6	0.526
Diabetes mellitus, *n* (%)	14 (37.8)	9 (23.7)	17 (42.5)	0.175
Hypertension, *n* (%)	17 (45.9)	24 (63.1)	21 (52.5)	0.426
Obstructive lung disease, *n* (%)	2 (0.5)	2 (0.5)	1 (0.2)	0.345
Operation time, min	189 ± 37	196 ± 44	198 ± 38	0.984
Surgery type				
CABG, *n* (%)	31 (83)	32 (84)	33 (0.82)	0.483
Valve replacement, *n* (%)	4 (10)	2 (5)	4 (10)	0.876
CABG and valve replacement, *n* (%)	2 (5)	4 (10)	3 (7.5)	0.724
IMA harvest, *n*				
Yes	33	32	33	0.347
No	4	6	7	
Fentanyl total dose, *µ*g	1320 ± 185.3	1383 ± 194.6	1302 ± 191.1	0.281

Data presented as mean ± SD unless otherwise indicated. CABG: coronary artery bypass graft; IMA: internal mammary artery; TENS: transcutaneous electrical nerve stimulation.

**Table 2:** Postoperative outcomes (0 h to 24 h).

Outcome	Treatment group			*P*
	Control (*n* = 37)	Parasternal block (*n* = 38)	TENS (*n* = 40)	
Morphine consumption, mg	52.4 ± 23.1	26.5 ± 13.34	37 ± 15.2	<0.001^*∗*^
Extubation time, min	217.8 ± 93	210.2 ± 85.4	193.6 ± 90.2	0.344
Length of ICU stay, h	24.4 ± 3.8	22.8 ± 5.5	23.7 ± 5.8	0.975
Length of hospital stay, days	5.8 ± 0.8	6.4 ± 0.9	6.2 ± 0.7	0.456
Tramadol consumption, mg	75.5 ± 55.5	50.5 ± 42.5	75.5 ± 45.5	0.445

Data presented as mean ± SD unless otherwise indicated. ^*∗*^ *P* < 0.05 considered to be statistically significant difference among groups. ICU: intensive care unit; TENS: transcutaneous electrical nerve stimulation.

## References

[1] Cheng D. C. H., Karski J., Peniston C. (1996). Morbidity outcome in early versus conventional tracheal extubation after coronary artery bypass grafting: a prospective randomized controlled trial. *Journal of Thoracic and Cardiovascular Surgery*.

[2] Higgins T. L. (1992). Pro: early endotracheal extubation is preferable to late extubation in patients following coronary artery surgery. *Journal of Cardiothoracic and Vascular Anesthesia*.

[3] Mueller X. M., Tinguely F., Tevaearai H. T., Revelly J.-P., Chioléro R., Von Segesser L. K. (2000). Pain location, distribution, and intensity after cardiac surgery. *Chest*.

[4] Konstantatos A., Silvers A. J., Myles P. S. (2008). Analgesia best practice after cardiac surgery. *Anesthesiology Clinics*.

[5] McDonald S. B., Jacobsohn E., Kopacz D. J. (2005). Parasternal block and local anesthetic infiltration with levobupivacaine after cardiac surgery with desflurane: the effect on postoperative pain, pulmonary function, and tracheal extubation times. *Anesthesia and Analgesia*.

[6] Power I. (2005). Recent advances in postoperative pain therapy. *British Journal of Anaesthesia*.

[7] Kocabas S., Yedicocuklu D., Yuksel E., Uysallar E., Askar F. (2008). Infiltration of the sternotomy wound and the mediastinal tube sites with 0.25% levobupivacaine as adjunctive treatment for postoperative pain after cardiac surgery. *European Journal of Anaesthesiology*.

[8] Solak O., Turna A., Pekcolaklar A. (2007). Transcutaneous electric nerve stimulation for the treatment of postthoracotomy pain: a randomized prospective study. *Thoracic and Cardiovascular Surgeon*.

[9] Baki E. D., Öz G., Kokulu S. (2015). Comparison of transcutaneous electrical nerve stimulation and paravertebral block for postthoracotomy pain relief. *Thoracic and Cardiovascular Surgeon*.

[10] Gregorini C., Cipriano G., de Aquino L. M., Rodrigues Branco J. N., Bernardelli G. F. (2010). Short-duration transcutaneous electrical nerve stimulation in the postoperative period of cardiac surgery. *Arquivos Brasileiros de Cardiologia*.

[11] Eljezi V., Dualé C., Azarnoush K. (2012). The analgesic effects of a bilateral sternal infusion of ropivacaine after cardiac surgery. *Regional Anesthesia and Pain Medicine*.

[12] Emmiler M., Solak O., Kocogullari C. (2008). Control of acute postoperative pain by transcutaneous electrical nerve stimulation after open cardiac operations: a randomized placebo-controlled prospective study. *The Heart Surgery Forum*.

[13] Fiorelli A., Morgillo F., Milione R. (2012). Control of post-thoracotomy pain by transcutaneous electrical nerve stimulation: effect on serum cytokine levels, visual analogue scale, pulmonary function and medication. *European Journal of Cardio-Thoracic Surgery*.

[14] Lima P. M., Farias R. T., Carvalho A. C. (2011). Transcutaneous electrical nerve stimulation after coronary artery bypass graft surgery. *Revista Brasileira de Cirurgia Cardiovascular*.

[15] Stubbing J. F., Jellicoe J. A. (1988). Transcutaneous electrical nerve stimulation after thoracotomy. Pain relief and peak expiratory flow rate-a trial of transcutaneous electrical nerve stimulation. *Anaesthesia*.

[16] Forster E. L., Kramer J. F., Lucy S. D., Scudds R. A., Novick R. J. (1994). Effect of TENS on pain, medications, and pulmonary function following coronary artery bypass graft surgery. *Chest*.

[17] Sbruzzi G., Silveira S. A., Silva D. V., Coronel C. C., Della Méa Plentz R. (2012). Transcutaneous electrical nerve stimulation after thoracic surgery: systematic review and meta-analysis of 11 randomized trials. *Revista Brasileira de Cirurgia Cardiovascular*.

[18] Solak O., Emmiler M., Ela Y. (2009). Comparison of continous and intermittant transcutaneus electrical nerve stimulation in postoperative pain management after coronary artery bypass grafting: a randomized, placebo-controlled prospective study. *The Heart Surgery Forum*.

